# Long-term outcome of cerebrospinal fluid diversion in patients with intracranial germinoma at Ramathibodi Hospital

**DOI:** 10.1007/s00423-025-03631-w

**Published:** 2025-02-14

**Authors:** Wasawat Muninthorn, Wattana Mahattanakul, Siriwut Pokanan, Atthaporn Boongird, Tanaporn Jaroenngarmsamer, Ake Hansasuta

**Affiliations:** Division of Neurosurgery, Department of Surgery, Faculty of Medicine Ramathibodi Hospital, Mahidol University, 270 Rama VI Road, Bangkok, 10400 Thailand

**Keywords:** Intracranial germinoma, CSF diversion, Endoscopic third ventriculostomy, Shunt, Hydrocephalus, Long-term outcome

## Abstract

**Introduction:**

Intracranial germinoma has a favorable prognosis with modern therapies, but the long-term outcome of cerebrospinal fluid (CSF) diversion for its associated hydrocephalus has been rarely focused on.

**Purpose:**

To evaluate the long-term success of CSF diversion methods—endoscopic third ventriculostomy (ETV) versus ventriculoperitoneal (VP) shunt—in intracranial germinoma patients.

**Methods:**

Only pure intracranial germinomas with obstructive hydrocephalus and a minimum follow-up duration of 24 months were retrospectively reviewed. Their demographics, as well as pre-and postoperative data, were recorded. Patients were stratified into the ETV and the non-ETV groups and subsequently compared to determine the longevity and morbidity related to the procedures. Factors associated with the failure of CSF diversion were examined.

**Results:**

From 1993 to 2022, eighty-three pathologically confirmed intracranial germinomas were identified. Excluding four cases of mixed pathology, eight with incomplete data, and two with insufficient follow-up, we enrolled 69 eligible patients for analysis. Among them, forty-three cases with obstructive hydrocephalus were classified into the ETV (*n* = 22) and non-ETV (*n* = 21) groups. No intraoperative or immediate postoperative complications occurred. With a median follow-up of 101 months (IQR 77.75–139.75), the ETV group had no failures. In the non-ETV cohort (median follow-up 144 months (IQR 97–210)), two VP shunt cases (9.5%) required revision due to blockage, and two patients (9.5%) experienced transient over-drainage. These 4 patients were without long-term difficulty despite short-term cumbersome events. No significant factors predicting CSF diversion failure were identified. To date, all 43 patients are alive without metastases, maintaining a good quality of life.

**Conclusion:**

This study highlights ETV as a preferred CSF diversion method in pure intracranial germinoma, achieving 100% success without morbidity. Apart from simultaneous biopsy, avoiding a separate operation, this approach eliminates shunt-related complications, ensuring long-term quality of life in patients with extraordinary prognoses from modern chemo- and radiotherapy.

## Introduction

Modern treatments for intracranial germinoma result in remarkable survival of the patients, unlike other central nervous system (CNS) tumors [1–5]. Interestingly, despite numerous oncology articles supporting its superb outcomes, the specific long-term appraisal of the cerebrospinal fluid (CSF) diversion in cases with obstructive hydrocephalus was lacking. The available literature offered various data on managing hydrocephalus in patients with germinoma; however, they each had strengths and limitations. Out of 153 histologically verified germ cell tumors, Matsutani et al. did not report the follow-up duration of their 63 pure germinomas (41.2%) nor the specific narration on their CSF-diversion management [1]. Hong et al. described the 127 CNS germ cell tumors classified into low- and high-risk groups. Although the details of initial hydrocephalus management for pure germinomas were provided in each stratified risk, unfortunately, their reported follow-up was a mixture of both cohorts. Furthermore, the study did not emphasize the long-term outcomes of different CSF-related procedures [4]. Schulz et al. focused on endoscopic third ventriculostomy (ETV) in pediatric pineal region tumors. Not only a few proportions (4 of 28) were germinomas, but this cohort also lacked the specific follow-up on the hydrocephalus for these 4 individuals [6]. Shono et al. reported survival, remission rates, and initial hydrocephalus management with ETV in 8 of their 12 pure germinoma patients. Like previously mentioned articles, the CSF-diversion failure or long-term outcomes were not demonstrated despite a mean follow-up duration of 78.6 months [7]. Although Ronsley et al. provided the most extended follow-up cohort (median = 10.4 years), they did not demonstrate specific details on the failures of their CSF-diversion procedures, two out of 22 cases of germinoma with hydrocephalus [8]. Like Hong et al., no pathological confirmation was obtained in every patient before chemotherapy [4, 8]. The studies above provided insights into initial hydrocephalus management in, for the most part, heterogeneous pathologies. Still, the sustainability of CSF-related surgeries and their failure in pure CNS germinoma cases remains undetermined.

Therefore, our primary goal was to exclusively examine the longevity of different CSF diversion techniques in patients with pure intracranial germinoma at our hospital. Associated risk factors leading to surgical failure were also assessed.

## Materials and methods

### Study design

After the institutional ethics committee approved this project, a comprehensive retrospective chart review from 1993 to 2022 was conducted. Patients with incomplete data or less than 24 months of follow-up were excluded. Histopathological confirmation of germinomatous germ cell tumors or germinomas must be available. Demographic, clinical, and radiographic data, as well as the surgery performed, were assessed. Intra- and postoperative complications were recorded. The need for repeat surgical intervention to treat CSF diversion failure after the initial surgery was recorded. Factors that might influence the outcome of CSF diversion were examined for statistical significance. Any sequelae would be identified after each surgical procedure, as well as the overall tumors’ treatment response, disease relapse, and new distant metastasis after initial therapy.

### Germinoma Diagnosis

Only histopathologically confirmed pure intracranial germinomas obtained at our institute were included in the study. Methods for tissue retrieval were based on each surgeon’s preference. The operations were either craniotomy for tumor resection (or biopsy), stereotactic percutaneous needle procedure, or endoscopic transventricular technique. To avoid possible mixture by non-germinomas, the included cases must have had serum alpha-fetoprotein (AFP) ≤ 10 nanograms per deciliter (ng/dL) and beta-human chorionic gonadotropin (β-hCG) ≤ 50 unit per liter (U/L) [2, 4, 9].

Every patient had contrast-enhanced magnetic resonance imaging (MRI) of the craniospinal axis to determine the status of leptomeningeal metastasis before starting their treatment. Radiographic evidence of such or positive cytology by lumbar puncture determined its spreading.

### Obstructive hydrocephalus and CSF diversion procedures

Only patients with symptomatic hydrocephalus caused by a confirmed germinoma were included in the study. Before the turn of the 21st century, the only CSF diversion available was ventricular shunting. Later, surgical methods were chosen based primarily on the tumor’s location and the surgeon’s preference. Some of our surgeons always performed ETV in cases with optimal anatomy and subsequently biopsied the tumor during the same operation utilizing a similar technique as previously described [10, 11]. Alternatively, ventriculoperitoneal (VP) shunts were implanted in patients whose ETV was considered unperformable due to anatomical limitations or individual surgeon expertise. Also, depending on the surgeons’ decision, some patients had shunt insertion before or after tissue diagnosis. Among them, some underwent shunt procedures as a separate surgery, while other shunts were inserted during the same operation with a tumor biopsy. Of note, a few patients referred from outside hospitals had VP shunts without tissue diagnosis. The common reasons were the lack of proper instruments or the inexperience of surgeons on duty. As a result, those shunts were already performed as life-saving measures before patients were later transferred to our institute. Thus, based on the included germinomas’ CSF diversion methods, they were divided into ETV versus standard VP shunt (non-ETV) groups.

### Chemo- and radiotherapy protocol

Prior to January 2000, the non-surgical treatment protocol was not uniform. After that, a new treatment paradigm of induced chemotherapy, described by Worawongsakul et al., became standard at our institute [5]. Etoposide, bleomycin, cisplatin, and carboplatin were commonly utilized as early as 14 days following the histopathology confirmation. MRI would be obtained to determine germinoma’s response to chemotherapy before starting radiation within 4 weeks after the completion of the 3rd cycle. For complete or near-complete tumor shrinkage, whole-ventricular radiotherapy for 21–24 Gray (Gy), 1.8–2 Gy daily, 5 fractions per week, with a primary tumor boost, up to a total dose of 30 Gy, was administered. Those with partial response after chemotherapy had higher radiation doses totaling 34–36 Gy. In cases with basal ganglia or thalamic locations, these germinomas received whole-brain radiotherapy with an intensified dose at the primary tumor site. Patients with spinal metastasis at the time of diagnosis underwent craniospinal irradiation with a local boost at the primary tumor area (30–36 Gy).

### Outcome and variable definition

After chemo- and radiotherapy, all patients were followed at our outpatient clinic by annual MRI scans. The survival of their CSF diversion was defined as the time from the initial ETV or VP shunt until patients developed symptoms/signs of raised intracranial pressure along with confirmation by radiographs, requiring another CSF diversion procedure due to the failure of the previous operation. When there was no such event, the duration would be until their latest follow-up visit or death if they did not need any revision. Possible factors associated with the failure of the CSF diversion methods were assessed.

The secondary outcomes were patients’ survival, disease relapse, and CSF diversion complications. The overall survival was defined as the time from diagnosis to the latest follow-up visit or death. The disease relapse-free survival was determined by the time of tissue diagnosis to the time of either confirmation of new tumor recurrence, new metastasis, or the latest follow-up visit without recurrence. Complications by CSF-related operations were also recorded. Our definition of over-drainage included the symptoms such as headache provoked by the upright posture, improved by recumbency, in association with radiographic signs such as slit ventricles, venous sinus engorgement, or subdural hematoma [12]. Metastasis was determined by the presence of a new tumor in one or more distant organs, such as the peritoneum, in VP shunt cases or craniospinal leptomeningeal spread in the ETV group.

### Statistical analysis

Data analysis was performed using STATA version 14.2 (STATA Corp., TX, USA). The record was analyzed among the groups. Categorical variables were evaluated using the Chi-square test or Fisher’s exact test, and the data were reported as numbers and percentages. Continuous data were described as mean ± standard deviation (SD), whereas non-continuous data were reported as median and interquartile ranges (IQR). For Continuous variables, comparisons using the Wilcoxon rank-sum (Mann-Whitney) test were conducted. Survival analysis using the Log-rank test was calculated. Uni- and multivariate analysis calculated possible factors that might influence failures of the initial CSF diversion. Differences were considered statistically significant when the p-value was less than 0.05 (2-sided).

## Results

For 30 years, eighty-three intracranial germinomas with confirmed pathology have been identified at Ramathibodi Hospital. After excluding 4 cases of mixed neoplasms, eight with incomplete data, and two patients with insufficient follow-up duration, there were 69 cases of unequivocal intracranial germinoma for our assessment. Fifty-four patients (78.3%) were male with a median age of 13 (IQR 12–19). Thirty-three (47.8%) tumors were in the pineal region; hence, Parinaud syndrome was, not surprisingly, the most common presenting sign in 35 cases (50.7%). With the median follow-up duration of 138 months (IQR 93–193), all 69 patients were alive at their last clinic visit.

Of the 69 pure intracranial germinomas, forty-three patients (62.3%) had obstructive hydrocephalus at presentation requiring CSF diversion. Table 1 compares the demographics of the 43 patients stratified into the ETV (*n* = 22) and non-ETV (*n* = 21) cohorts. While there were no significant differences between the groups regarding age, gender, clinical presentation, β-hCG, or tumor size, the pineal location was more common in the patients who underwent ETV. At the time of diagnosis, this group of patients also had higher serum AFP and more frequent spinal drop metastasis. Among the 21 cases in the non-ETV group, seven patients (33.3%) had VP shunt placement, without tissue diagnosis, at outside hospitals before referral. The rest (*n* = 14, 66.7%) underwent post-craniotomy shunt insertion in the same setting or as a separate procedure at our institute. As for the tissue retrieval route in this cohort, twenty had craniotomies, and one patient underwent a stereotactic needle biopsy. In contrast, all patients in the ETV group had tumor biopsy after CSF bypass at the same operation setting.

**Table 1:** Demographic data of intracranial germinoma patients with obstructive hydrocephalus after undergoing initial CSF-diversion operation (*n* = 43)

Characteristics	Total (*n* = 43)	Non-ETV (*n* = 21)	ETV (*n* = 22)	*p*-value
**Age (years);** median (IQR)	14 (12–22)	13 (12–16)	17.5 (13–23)	0.134
**Sex;** n (%)				
Male	40 (93.0)	19 (90.5)	21 (95.4)	0.607
Female	3 (7.0)	2 (9.5)	1 (4.6)	
**Presentation;** n (%)				
Drowsiness	1 (2.3)	0 (0.0)	1 (4.6)	> 0.999
Seizure	4 (9.3)	3 (14.3)	1 (4.6)	0.345
Visual disturbance	5 (11.6)	3 (14.3)	2 (9.1)	0.664
Headache	28 (65.1)	11 (52.4)	17 (77.3)	0.087
Parinaud syndrome	33 (76.7)	15 (71.4)	18 (81.8)	0.420
Panhypopituitarism	10 (23.3)	5 (23.8)	5 (22.7)	0.933
Diabetes insipidus	10 (23.3)	6 (28.6)	4 (18.2)	0.420
Others	2 (4.7)	1 (4.8)	1 (4.6)	> 0.999
**Tumor location;** n (%)				
Sellar/Suprasellar	2 (4.7)	2 (9.5)	0 (0.0)	0.233
Pineal	31 (72.1)	12 (57.1)	19 (86.4)	**0.033**
Basal ganglia/Thalamus	1 (2.3)	1 (4.8)	0 (0.0)	0.488
Bi-focal lesions	9 (20.9)	6 (28.6)	3 (13.6)	0.281
**Tumor diameter (cm.);** mean ± SD	2.8 ± 1.0	2.6 ± 1.1	3.1 ± 0.9	0.129
**β-hCG serum level;** n (%)				
< 1.1 U/L	29 (87.9)	16 (88.9)	13 (86.7)	0.846
≥ 1.1 U/L	4 (12.1)	2 (11.1)	2 (13.3)	
**AFP serum level (ng/dL); ** median (IQR)	2.6 (1.0–1.7)	1.1 (0.9–2.1)	2.0 (1.5–3.6)	**0.029**
**Initial metastasis status; ** n (%)	6 (14.0)	1 (4.8)	5 (22.7)	0.185
**Positive cytology without imaging evidence;** n (%)	3 (7.0)	1 (4.8)	2 (9.1)	> 0.999
**Intracranial metastasis;** n (%)	1 (2.3)	1 (4.8)	0 (0.0)	0.488
**Spinal drop metastasis;** n (%)	5 (11.6)	0 (0.0)	5 (22.7)	**0.048**

Abbreviations: ETV, endoscopic third ventriculostomy; CSF, cerebrospinal fluid; IQR, interquartile range; SD, standard deviation; AFP, beta serum alpha-fetoprotein; β-hCG, beta-human chorionic gonadotropin; ng/dL, nanogram per deciliter; U/L, units per liter

**Table 2:** Treatment outcomes and complications of intracranial germinoma patients with obstructive hydrocephalus after undergoing initial CSF-diversion operation (*n* = 43)

Characteristics	Total (*n* = 43)	Non-ETV (*n* = 21)	ETV (*n* = 22)	*p*-value
**Follow-up duration (months);**				
mean ± SD	130.2 ± 65.6	150.4 ± 77.8	111.0 ± 45.3	
(Range)	(24–288)	(24–288)	(30–196)	**0.048**
median	130	144	101	
(IQR)	(79.5–171)	(97–210)	(77.75–139.75)	
**Disease relapse; ** n (%)	0 (0)	0 (0)	0 (0)	-
**Total complications; ** **n (%)**	4 (9.3)	4 (19)	0 (0)	**0.0485**
**Infection;** n (%)	0 (0)	0 (0)	0 (0)	-
**Over drainage syndrome;** n (%)	2 (4.7)	2 (9.5)	0 (0)	0.233
**CSF diversion failure;** n (%)	2 (4.7)	2 (9.5)	0 (0.0)	0.233
**New spinal drop metastasis;** n (%)	0 (0)	0 (0)	0 (0)	-
**Peritoneal metastasis;** n (%)	0 (0)	0 (0)	0 (0)	-

Abbreviations: ETV, endoscopic third ventriculostomy; CSF, cerebrospinal fluid; IQR, interquartile range; SD, standard deviation

**Fig. 1:**
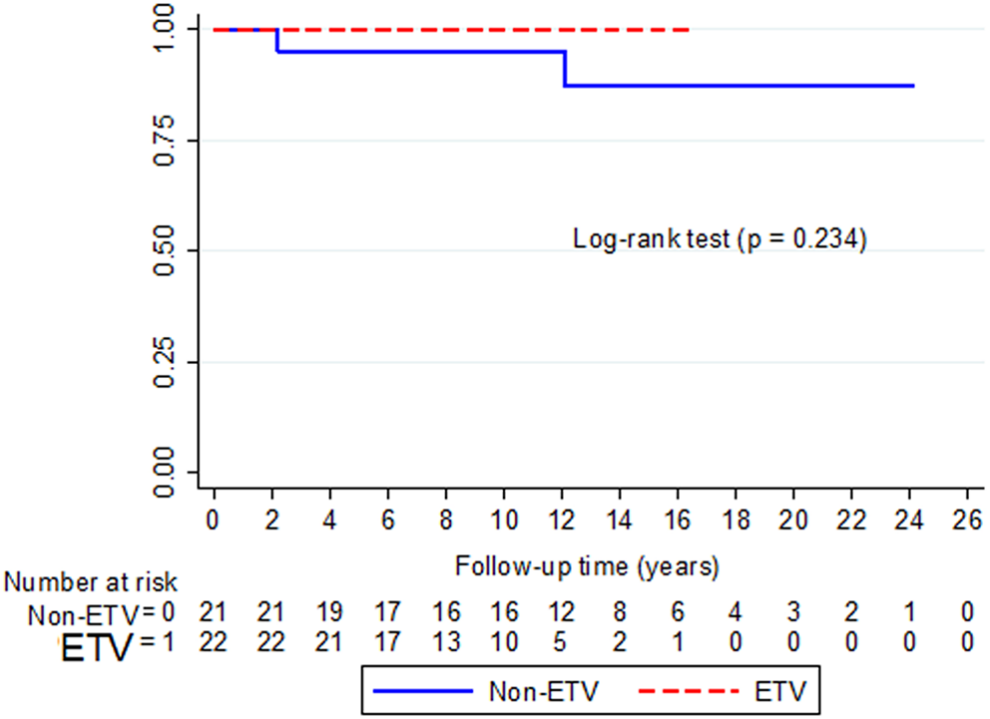
Additional-CSF-intervention-free survival compared between the ETV and non-ETV groups

The non-ETV cohort had a median follow-up duration of 144 months (IQR 97–210), and the overall shunt failure was 9.5% (2 of 21). (Table 2) These two suffered a malfunction at 26.4 months and 145.2 months after the VP shunts. With hardware revisions, the patients resumed their everyday lives until the present. Moreover, two other cases (9.5%) in the non-ETV group developed symptoms and radiographic signs of shunt over drainage. Fortunately, their thin subdural hematoma and intracranial hypotension-type headache eventually resolved without surgical intervention after 3 and 6 months. In contrast, the ETV group, with a median follow-up duration of 101 months (IQR 77.75–139.75), had no failure or surgically related complication. However, the difference in CSF-diversion survival did not reach statistical significance (*p* = 0.223), as shown in Fig. 1. But on the other hand, the combined complication rate of 19% (4 of 21) must not be overlooked (*p* = 0.048). Despite the aforementioned shunt-related complications, these 43 cases were alive, enjoying a good quality of life to date. At all patients’ last follow-up, no peritoneal metastasis was observed in the non-ETV group, and no new spinal drop metastasis was found in the ETV cases. Furthermore, the examination of possible factors related to shunt failure did not detect any relevant variable through uni- or multivariate analysis (Table 3).

**Table 3:** Factors associated with CSF diversion failure in the non-ETV group

Variable	Univariate analysis		Multivariate analysis	
	OR (95% CI)	*p*-value	OR (95% CI)	*p*-value
**Age** (years)	1.02 (0.82–1.28)	0.849	0.86 (0.25–2.63)	0.933
**Sex**	-		-	
Male				
Female				
**Presentation**				
Drowsiness	-		-	
Seizure	-		-	
Visual disturbance	9.25 (0.48–178.07)	0.140	8.27 (0.25–68.01)	0.098
Headache	-		-	
Parinaud syndrome	-		-	
Panhypopituitarism	-		-	
Diabetes insipidus	-		-	
Other	-		-	
**β-hCG serum level**	-		-	
< 1.1 U/L				
≥ 1.1 but less than 50 U/L				
**AFP serum level** (ng/dL)	0.99 (0.85–1.13)	0.841	1.01 (0.88–1.15)	0.926
**Follow-up duration** (months)	0.99 (0.97–1.02)	0.934	0.99 (0.86–1.13)	0.998
**Tumor location**				
Sellar/Suprasellar	-		-	
Pineal	0.72 (0.03–13.45)	0.831	0.67 (0.02–12.01)	0.977
Basal ganglia/Thalamus	-		-	
Bi-focal lesions	2.80 (0.14–53.71)	0.495	2.10 (0.05–33.85)	0.845
**Tumor diameter** (cm)	0.98 (0.26–3.79)	0.982	0.98 (0.18–5.41)	0.990

Abbreviations: OR, odd ratio; CI, confidence interval; AFP, beta serum alpha-fetoprotein; β-hCG, beta-human chorionic gonadotropin; ng/dL, nanogram per deciliter; U/L, units per liter; cm, centimeter

## Discussion

An excellent prognosis, up to 85–95% survival, can be achieved with a modern chemo-radiation treatment regimen for pure intracranial germinomas [1–5]. Drastic shrinkage of this kind of malignancy can be frequently seen after one or two cycles of chemotherapy. Therefore, surgical tumor resection does not play a significant role in its management except for tissue biopsy and CSF diversion [1, 2]. In contrast to the ample evidence confirming medical treatment’s efficacy, there was a substantial lack of literature concerning the long-term CSF drainage issue in such a disease [3, 4, 6, 8]. Therefore, we retrospectively explored this particular matter to determine the longevity of different methods so as to discern the procedure of choice for dealing with pure intracranial germinoma.

To eliminate the influence of the different natural histories of other types of neoplasms, histopathological confirmation of germinoma was mandatory for all patients in this study. In addition, our protocol was that before initiating chemo- and radiotherapies at Ramathibodi Hospital, types and subtypes of neoplasm must be definitive [5]. Furthermore, based on the study by Shono et al., mixed pathology in patients with an initial impression of classic intracranial germinoma by radiographs was not uncommon [7]. Hence, unlike other series, our cohort was an uncontaminated group of indisputably pure germinomas.

Our patients’ demographic data were comparable with the previously published articles [1–4]. Male adolescents, typically younger than 20 years old, common pineal region and Parinaud syndrome at presentation were typical. However, our obstructive hydrocephalus incidence (62.3%) was slightly above the reported range of 42.8–56% [3, 8, 13]. Although Hong et al. published results from a larger patient cohort with relatively extensive follow-up (median = 8.4 years), the authors did not specifically analyze their CSF diversion outcomes. Moreover, their mixed pathologies made interpreting the results challenging [4]. Another study, with a mean follow-up time of 124 months, mentioned temporary and permanent CSF diversion. However, not all of their germinomas were diagnosed by histopathology [8]. In contrast to the articles mentioned above, our study comprised only the genuine intracranial germinoma, all by tissue biopsy confirmation plus strict AFP/ β-hCG criteria, to avoid the influence of various neoplasms affecting outcomes. In addition, our median follow-up time of 138 months was the longest ever reported.

With no intraoperative complications in our 43 patients, the non-ETV group had significantly longer follow-up time. This was essentially because VP shunt had been the backbone long before the beginning of ETV after the millennium. Our data demonstrated the propensity for ETV in managing the pineal region tumors since it was the method of choice on this site. Endoscopic transventricular access not only achieves CSF bypass but also obtains specimens simultaneously [7, 8]. In contrast, almost half of the non-ETV group had two separate surgeries at different points in time. There was no immediate re-operation due to recurring obstructive hydrocephalus or surgically related infection; however, two shunted patients (9.5%) experienced transient over-drainage. Both were lucky to have had spontaneous resolution without surgical intervention. Despite the two suffering non-life-threatening events, all 43 patients proceeded with chemotherapy after surgeries.

Regarding the long-term success of the two different CSF diversion procedures, the ETV group achieved 100% patency, while two shunt revisions (9.5%) in the non-ETV patients were necessary. Hence, the combined short- and long-term complications in the non-ETV group were statistically more frequent compared to the ETV group (*p* = 0.048). One can simply realize what these four individuals must go through, i.e., additional pain, running the risks of infection or hemorrhage and dealing with the healthcare cost, time lost for their work or living, and potential future shunt malfunctions. These disadvantages are not negligible; in particular, those with pineal region tumors could have had minimal to no associated risks had they undergone ETV instead of shunts. Other than the longevity of CSF bypass, previously published articles found that intracranial germinomas can disseminate via CSF flow within the subarachnoid space. A hypothesis is that an acquired stoma connecting the third ventricular floor to the prepontine cistern by ETV may portray an increased risk of developing new spinal drop metastasis [6, 7]. In spite of significantly more cases with preexisting spinal seedings prior to surgical interventions, we found no such new incidence in the ETV cohort. Moreover, up to their last follow-up, the pretreatment spinal spreads were not discovered again. In addition, there was no peritoneal metastasis in any VP shunt cases. At the time of this data analysis, all 43 patients were alive and living well.

Despite our stringent pathological and laboratory criteria for diagnosing these pure intracranial germinomas and their lengthy follow-ups, this retrospective data review had a few pitfalls. First, the eight cases with incomplete data combined with the 2 insufficient follow-up durations could have somewhat impacted the results in one way or another. Secondly, there was no strict protocol for the CSF diversion technique. Different surgeons had their preferences for managing this condition. Even after the millennium, the beginning of ETV at our institute, shunts were still performed, up to now, in the otherwise perfect candidates for ETV with biopsy, such as pineal location tumor. These shunted cases will always be at risk for malfunction; hence, the blockage rate can even be higher than already reported with longer follow-up duration. Thirdly, we have not carried out the algorithm, per a previous publication by Ronsley et al., in which, occasionally, histological diagnosis was not required and CSF diversion, in some cases, was only temporarily necessary [8]. Our hospital has limitations that prevent us from implementing their patient care process. In actual practice, our main hurdle is the policy that histopathology must be definitive before chemotherapy; hence, every patient must have a biopsy. The limited bed quota for neurosurgery admission is another discernable factor, and any time-consuming stay of a patient with an external ventricular drain receiving chemotherapy after a definitive tissue interpretation would significantly affect others needing neurosurgical care. Hence, if these hindering issues are resolved, we enthusiastically expect to adopt Ronsley et al.’s protocol, bypassing definitive pathology in some cases before medical treatment begins. As a result, definitive CSF diversion can sometimes be avoided [8].

In summary, because intracranial germinoma, especially the pure type, has a miraculously extended life expectancy despite its malignant histology, neurosurgeons are responsible for ensuring that the patient’s quality of life after all treatments is not jeopardized by the choice of CSF diversion. In centers with capabilities for ETV, it should be prioritized over shunts due to this ideal bypass’s long-term sustainability and low-to-no risks.

## Conclusions

Our evidence, with an extensive follow-up period, supports ETV’s remarkable efficacy and superiority over the shunting procedure in dealing with obstructive hydrocephalus by pure intracranial germinoma. ETV with simultaneous biopsy should be strongly considered as the procedure of choice, particularly for the pineal location.

## Data Availability

No datasets were generated or analysed during the current study.

## Abbreviations

CSF: Cerebrospinal fluid
AFP: Alpha-fetoprotein
β-hCG: Beta-human chorionic gonadotropin
ETV: Endoscopic third ventriculostomy
VP: Ventriculoperitoneal
